# Home Characteristics as Predictors of Bacterial and Fungal Microbial Biomarkers in House Dust

**DOI:** 10.1289/ehp.1002004

**Published:** 2010-10-22

**Authors:** Joanne E. Sordillo, Udeni K. Alwis, Elaine Hoffman, Diane R. Gold, Donald K. Milton

**Affiliations:** 1 Channing Laboratory, Department of Medicine, Brigham and Women’s Hospital, Boston Massachusetts, USA; 2 Harvard Medical School and; 3 Harvard School of Public Health, Boston, Massachusetts, USA; 4 University of Maryland, College Park, Maryland, USA

**Keywords:** bacteria, dampness, fungi, home characteristics, indoor exposure, respiratory health

## Abstract

**Background:**

Measurement of fungal and bacterial biomarkers can be costly, but it is not clear whether home characteristics can be used as a proxy of these markers, particularly if the purpose is to differentiate specific classes of biologic exposures that have similar sources but may have different effects on allergic disease risk.

**Objective:**

We evaluated home characteristics as predictors of multiple microbial biomarkers, with a focus on common and unique determinants and with attention to the extent of their explanatory ability.

**Methods:**

In 376 Boston-area homes enrolled in a cohort study of home exposures and childhood asthma, we assessed the relationship between home characteristics gathered by questionnaire and measured gram-negative bacteria (GNB) (endotoxin and C10:0, C12:0, and C14:0 3-hydroxy fatty acids), gram-positive bacteria (GPB) (*N*-acetyl muramic acid), and fungal biomarkers [ergosterol and (1→6) branched, (1→3) β-d glucans] in bed and family room dust.

**Results:**

Home characteristics related to dampness were significant predictors of all microbial exposures; water damage or visible mold/mildew in the home was associated with a 20–66% increase in GNB levels. Report of cleaning the bedroom at least once a week was associated with reduced GNB, GPB, and fungi. Presence of dogs or cats predicted increases in home bacteria or fungi. The proportion of variance in microbial biomarkers explained by home characteristics ranged from 4.2% to 19.0%.

**Conclusions:**

Despite their associations with multiple microbial flora, home characteristics only partially explain the variability in microbial biomarker levels and cannot substitute for specific microbial measurements in studies concerned with distinguishing effects of specific classes of microbes.

Evaluation of home characteristics by questionnaire is less costly than measurement of multiple microbial flora in the home. Home characteristics, particularly those associated with increased moisture, have been linked to respiratory symptoms (Douwes et al. 1999; Fung and Hughson 2003; Garrett et al. 1998; Handal et al. 2004). Although it is known that increased dampness promotes the growth of microbial flora, the relationship of home characteristics to bacterial and fungal levels is imperfectly understood. Better understanding of that relationship can aid in evaluating the extent to which assessment of home characteristics can be a surrogate for measurement of bacteria and fungi in health effect studies, differentiating between types of microbial exposure, and identifying potentially modifiable conditions that may be the source of multiple exposures having protective or adverse health effects. Few studies have evaluated the relation of home characteristics to measures of multiple microbial exposures. Such an evaluation becomes increasingly important as we recognize that specific microbial agents from similar sources may differ in their effects. For example, we have shown that increased fungal exposures in the first year of life may increase the risk of allergic rhinitis (Stark et al. 2005), whereas endotoxin exposure may be a protective factor (Celedon et al. 2007; Litonjua et al. 2002).

Previously we demonstrated that sources of home dampness (humidifier use, water damage) predicted elevated levels of endotoxin, a gram-negative bacterial (GNB) biomarker, in the homes of infants enrolled in the Epidemiology of Home Allergens and Asthma Study (Park et al. 2001b). In this follow-up study of these children at school age (mean age, 7 years), we assessed the relation of home characteristics (questionnaire data and allergen levels) and demographics to measures of multiple microbial exposures. We evaluated the explanatory power and positive and negative predictive values (PPV and NPV, respectively) of home characteristics in their associations with GNB, gram-positive bacteria (GPB), and fungi.

## Materials and Methods

### Study cohort

The Epidemiology of Home Allergens and Asthma Study is an ongoing longitudinal birth-cohort study of the effects of environmental exposures on the risk of allergy and asthma in children born to parents with histories of allergies and/or asthma (Gold et al. 1999). The study was approved by the institutional review board of Brigham and Women’s Hospital. We obtained written informed consent from the primary caregivers. Screening and recruitment of the families was conducted between September 1994 and June 1996. A detailed description of subject recruitment and early-life sampling for endotoxin and allergens in the home has been published previously (Gold et al. 1999; Park et al. 2001a). Briefly, families from metropolitan Boston, Massachusetts, were recruited at a major Boston hospital immediately after the birth of the index child. Exclusion criteria were gestational age < 36 weeks, congenital abnormalities, hospitalization in the neonatal intensive care unit, maternal age < 18 years, and a plan to move within the next year.

### Home visits and microbial biomarker sample collection

When the index children were of school age (mean age, 7 years), we conducted a home visit and collected three dust samples, two from the family room and one from the index child’s bed. We used a Eureka Mighty-Mite vacuum cleaner (model 3621; Eureka Co., Bloomington, IN) modified to hold 19 × 90 mm cellulose extraction thimbles. For bed dust, all layers of the bedding were vacuumed for a total of 10 min. For family room dust samples, we vacuumed both a 1-m^2^ area of the family room floor for 2 min and an upholstered chair commonly used by the index child for 3 min. We repeated this procedure to collect a second family room dust sample. Of the 382 homes visited, 376 had microbial biomarker assessment in at least one room. Microbial biomarker levels were measured in 354 family room samples and in 299 beds. Family room dust samples were analyzed first for allergen levels and then for microbial biomarkers. If the first family room dust sample was consumed by allergen measurement (*n* = 49), the second dust sample was used for microbial biomarker assessment. In a subset of the cohort (*n* = 29), microbial biomarker measurements were performed on both the first and second family room samples. Paired *t*-tests indicated no significant difference between the two samples taken from the family room.

We administered a detailed questionnaire about home characteristics, including dampness-related variables (type of building, use of humidifier and dehumidifier, mold, water damage, central air conditioning), socioeconomic status (SES; income, race/ethnicity), carpeting, pests, frequency of cleaning (number of times the bedroom was cleaned per week), and number and sex of the children in the household. Exposure to pets (cats, dogs) and cockroaches was assessed by questionnaire and by allergen quantification in house dust.

### Dust samples

Within 24 hr after dust collection, we weighed and sieved the dust through a 425‐μm mesh sieve. We reweighed the fine dust and made aliquots for allergen and microbial marker analysis. Allergen measurements were given first priority in bed and family room dust. Of the 382 homes visited, 354 (93%) of the homes had sufficient family room dust and 294 (77%) had sufficient bed dust for microbial biomarker assessment.

### Microbial biomarker assays

We determined endotoxin bioactivity using both kinetic *Limulus* amebocyte lysate (LAL) and recombinant factor C (rFC) (Alwis and Milton 2006) assays with the kinetic *Limulus* assay with resistant parallel line estimation (KLARE) method as previously described (Milton et al. 1992). LAL and rFC reagents were obtained from Cambrex (Walkersville, MD), reference standard endotoxin from U.S. Pharmacopoeia, Inc. (Rockville, MD), and control standard endotoxin from Associates of Cape Cod (Woods Hole, MA). We assayed dust samples for 3‐hydroxy fatty acids (3-OHFAs) as biomarkers of lipopolysaccharide using a method described elsewhere (Park et al. 2004). The 3-OHFAs with the highest correlation to endotoxin (C10:0, C12:0, and C14:0, or “midchain length”) were summed and reported as picomoles per milligram of dust.

To measure peptidoglycan, we quantified the component *N*-acetyl muramic acid (2-acetamido-3-*O*-[(*R*)-1-carboxyethyl]-2-deoxy-d-glucose; hereafter muramic acid) by a previously described gas chromatography/mass spectrometry (GC/MS) method, using ^13^C-labeled cyanobacteria as an internal standard (Sebastian et al. 2004). Muramic acid levels mainly represent GPB (the peptidoglycan layer is approximately 10–15 times thicker in GPB than in GNB cell walls). Results were reported as nanograms of muramic acid per milligram of dust.

For fungi, we measured the biomarkers (1→6) branched (1→3)β-d-glucan (hereafter β-d-glucan) and ergosterol. For β-d-glucan measurement we used an enzyme-linked immunosorbent assay (Milton et al. 2001), and for ergosterol we used a previously described GC/MS method (Axelsson et al. 1995; Sebastian and Larsson 2003). Results were reported as nanograms per milligram of dust.

### Data analysis

Microbial biomarkers showed right-skewed distributions. Therefore, we performed log_10_ transformation of those measurements to obtain symmetrical, approximately Gaussian distributions. We used log-transformed data for analyses of microbial biomarkers as continuous outcomes. For both bed and family room dust, these microbial biomarkers included ergosterol level (nanograms per milligram of dust), β-d-glucan (nanograms per milligram of dust), midchain (C10:0, C12:0, C14:0) 3-OHFAs (picomoles per milligram of dust), endotoxin (endotoxin units per milligram of dust, by both LAL and rFC), and muramic acid (nanograms per milligram of dust). We created binary predictor variables using the data from the home characteristics questionnaire. For bed and family room biomarker models these predictors were dampness-related characteristics (type of building, use of humidifier and dehumidifier, visible mold/mildew, water damage, central air conditioning), pets (current cat ownership, dog ownership), pests (cockroach and mouse sightings in the past year), and carpeting (a potential reservoir for many microbial organisms). To avoid entering two collinear predictors in models, we created a composite variable for water damage and visible mold/mildew (water/mold index), equal to 1 for those with either or both home characteristics and 0 for those with neither. For bed dust biomarker models specifically, we also examined the influence of more than four stuffed animals on the bed, cleaning the bedroom once or more per week, and child’s sex on microbial biomarker levels in mattress dust. Additionally, variables for a continuously burning pilot light and the total number of household children (total boys and total girls) were considered as predictors of family room biomarker levels.

To build multiple regression models for bed and family room microbial biomarkers, we first constructed reduced models containing *a priori* home characteristics (those identified from the literature) and those statistically significant (*p* < 0.05) in univariate models. Home characteristics that were significant additions to the reduced model (*p* < 0.05) were entered into the multiple regression model. After selection of home characteristics, we adjusted final models for seasonal effects. Multiple regression models incorporating allergen levels (≥ 20 μg/g Can f 1, ≥ 8 μg/g Fel d 1, detectable Bla g 1) in the place of questionnaire data (current dog ownership, cat ownership, cockroach sightings in the past year) are also shown.

In secondary analyses, predictive values of individual home characteristics as indicators of GNB, GPB, and fungi were also assessed for bed exposure. To perform these calculations, microbial biomarker levels were classified as either above or below the median. *A priori* we chose the median as the cut-point because we have demonstrated that microbial exposures above this threshold have been associated with respiratory health (Litonjua et al. 2002; Park et al. 2001a). We computed PPVs and NPVs to express the probability of being above or below the median level of microbial exposure, given the presence or absence of a particular home characteristic. [PPV is calculated by determining the percentage of homes with an elevated microbial biomarker level (i.e., > median ergosterol) among all homes with a given home characteristic (i.e., “dog ownership” or > 20 μg/g Can f 1). NPV is calculated by determining the percentage of homes with decreased microbial biomarker levels (i.e., < median ergosterol) among all homes lacking a given home characteristic (i.e., < 20 μg/g Can f 1)]. In predictive value calculations for GNB, elevated exposure was defined as greater than the median level of endotoxin (LAL) or midchain 3-OHFAs in bed dust. To evaluate whether the cut-point influenced the overall inference regarding the PPV and NPV of the home characteristics for microbial markers, we also examined a second cut-point at the 75th percentile.

## Results

### Characteristics and correlations

Only a small percentage of the population reported living in an apartment, earning < $35,000/year, or using a dehumidifier or humidifier in the family room (Table 1). Other home characteristics such as dog or cat ownership, central air conditioning, mouse sightings, visible mold/mildew and/or water damage, and frequent cleaning were more prevalent (21.5–54.4%). Biomarker levels were higher in family room dust samples than in bed dust samples (Table 2). Correlations between midchain 3-OHFAs and endotoxin were moderate in both bed and family room dust samples (Table 3) (Pearson *r* = 0.39–0.45). Midchain 3-OHFAs were moderately correlated with β-d-glucan and ergosterol in the bed samples (*r* = 0.42 and 0.43) but were not as highly correlated in family room dust (*r* = 0.23 and 0.28). Muramic acid was most highly correlated with midchain 3-OHFAs. The fungal biomarkers ergosterol and β-d-glucan showed only a modest correlation in bed (*r* = 0.32) and family room (*r* = 0.22) samples.

### Categories of predictors

Predictors fell into broad categories: dampness, pets, socioeconomic factors, cleaning, and number/sex of children in the household. For multiple regression models, Tables 4 and 5 show the percent change in microbial biomarker levels associated with these predictors. Multiple regression models explained a low to moderate portion of the variability in microbial biomarker levels (*R*^2^ = 4.2–19.0%).

Characteristics related to dampness were the most consistent predictors of microbial biomarkers. The water/mold index predicted increased GNB biomarkers in both bed and family room dust. This variable was also associated with higher levels of the fungal biomarker ergosterol found in family room dust. Central air conditioning, a possible indicator of drier home conditions, predicted reductions in levels of GNB (endotoxin and midchain 3-OHFAs), GPB (muramic acid), and fungi (ergosterol and β-d-glucan). Living in an apartment, also associated with drier conditions, was a predictor of decreased fungal biomarkers in family room and bed dust.

Cleaning the bedroom at least once a week showed associations with lower levels of GNB (LAL endotoxin and midchain 3-OHFAs), GPB (muramic acid), and fungi (ergosterol). We also observed a similar trend for cleaning for endotoxin by rFC, but it did not achieve statistical significance in multiple regression models.

Although sources of dampness in both the bedroom and the family room predicted increases in all of the microbial biomarkers, the associations of pets with these biomarkers were less consistent. Regardless of whether ascertained by questionnaire or allergen level measurement, pets or pests had the same direction of association with levels of measured microbial biomarkers. However, in the bedroom, compared with reports of owning pets, high levels of dog and cat allergen were more strongly predictive of elevated GNB biomarkers. The direction of the association of dog with markers of mold differed by room, with dog predicting higher β-d-glucan levels in the bedroom but lower ergosterol and β-d-glucan levels in the family room. Black race/ethnicity was consistently associated with lower levels of microbial biomarkers, a predictor of lower GNB (rFC endotoxin) and fungi (β-d-glucan). Low income was associated with lower ergosterol levels but was not associated with other microbial biomarker levels.

For family room exposures, boys were associated with moderate increases in GNB, whereas girls were weakly associated with lower levels of GPB and fungi (β-d-glucan). However, the index child’s sex was not a significant predictor of microbial biomarkers in individual bed dust samples.

### Predictive values for home characteristics

We employed home characteristics from the strongest categories of predictors as tests for high (> median) microbial biomarker levels. Elevated pet allergens, home dampness, and infrequent cleaning were associated with a 71–75% probability (PPV) of increased (> median) GNB. PPVs observed for pet allergen levels as predictors of elevated GPB and fungi were substantially lower (PPV, 54–66%). NPVs, or the probability of microbial exposure at less than the median threshold given the absence of a home characteristic, ranged from 36% to 54% for all microbial biomarker levels [see Supplemental Material, Table 1 (doi:10.1289/ehp.1002004)]. Cut-point strongly influenced the predictive values of home characteristics. A cut-point at greater than the 75th percentile increased the NPV of dampness, pet, and cleaning characteristics to ≥ 70% [see Supplemental Material, Table 2 (doi:10.1289/ehp.1002004)].

## Discussion

The association between home characteristics (particularly dampness) and respiratory health is thought to be mediated by exposures to bacteria and fungi. Although many studies have examined the link between home environment and individual microbial biomarkers (i.e., endotoxin), this work simultaneously assessed the associations between home characteristics and the microbial milieu (GNB, GPB, and fungi). Our findings suggest the following: Presence of damp environment, pets, and less frequent cleaning partially explain higher levels of home bacteria and mold; sources of microbial exposures (represented by home characteristics) overlap for GNB, GPB, and fungi and cannot be used to distinguish between these groups of organisms.

### Categories of predictors

Four categories of predictors [dampness, pets, cleaning, and demographics (SES and sex)] were consistently associated with the home microbial environment. For GNB, GPB, and fungi, indoor home characteristics associated with moisture were the strongest predictors in multiple regression models. The link between dampness and GNB has been shown in previous studies, where water damage and high humidity conferred elevated levels of endotoxin or 3-OHFAs (Hyvarinen et al. 2006; Park et al. 2000; Wickens et al. 2003). The inverse relationship between GNB levels and central air conditioning, a potential indicator of dryness, reported in the present work supports a similar published association (Gereda et al. 2001). Numerous studies have also identified moisture as a promoter of increased fungal levels in the indoor environment (Douwes et al. 1999; O’Connor et al. 2004). The causes and consequences of dampness itself are heterogeneous in nature, and moisture damage may involve a variety of different building materials. Although dampness has been shown to increase GNB, GPB, and fungi, the characteristics of microbial colonization (e.g., bacteria or fungi, taxa, genus, species) resulting from damp conditions will depend on the nature of the individual moisture problem (Nevalainen and Seuri 2005). Although indicators of home dampness may relate to total microbial burden, they cannot provide insight into the specific type of microbial growth.

Pet ownership and pet allergen levels were positively associated with higher levels of fungi (β-d-glucan) and GNB (midchain 3-OHFAs and endotoxin). The importance of pets as contributors to the microbial environment has been documented in other studies (Bischof et al. 2002; Gehring et al. 2001; Gereda et al. 2001; Heinrich et al. 2001; Thorne et al. 2009). In the present work, dog exposure was not associated with endotoxin, even though dog ownership was a strong predictor of this GNB biomarker in a previous analysis of this cohort (Park et al. 2001a, 2001b). It is possible that more detailed questions about the dog’s activity (where the dog was permitted inside the household, whether it often went outside) would have refined this home characteristic, lending more power to detect a link between dogs and house dust endotoxin in the present work. Cat allergen levels were associated with bed dust endotoxin, a finding consistent with other studies (Giovannangelo et al. 2007). However, cat ownership was not associated with increased GNB, suggesting that cat allergen measurement is actually a better indicator of pet presence in the sampling location. It may be better because *a*) allergen may be brought into the household even when the participants do not own the pet, or *b*) allergen and microbes associated with pets may vary depending on whether or not the pets are allowed in the bedroom. Nevertheless, report of a pet is generally closely correlated with high pet allergen levels, whereas (as we have previously reported) sightings of pests are poorly correlated with allergen levels for either cockroach or mice (Chew et al. 1998).

Next to sources of moisture, cleaning frequency was the most consistent variable associated with the microbial environment in the home. Cleaning the bedroom at least once a week was related to decreased levels of fungi, GPB, and GNB. Lower cleaning frequency (vacuuming and dusting) has been associated with increased levels of β-d-glucan and 3-OHFAs in other reports (Bischof et al. 2002; Gehring et al. 2001; Hyvarinen et al. 2006). The potential for an individual to alter his or her home microbial exposure, perhaps as a result of experiencing allergy symptoms, should be accounted for in epidemiologic studies on home exposures and allergic disease. Because an individual s own commensal flora has been shown to affect the composition of house dust (Täubel et al. 2009) and could potentially vary by sex, we examined sex as a predictor in multiple regression models. Although sex was not associated with microbial biomarker levels in the bedroom, the number of boys was positively associated with bacteria and fungal markers in the family room, perhaps because of differential behaviors by sex (Else-Quest et al. 2006).

In this cohort, low income level was linked to decreased levels of fungal exposure (ergosterol). Race/ethnicity was associated with reductions in GNB (endotoxin) and fungi (β-d-glucan), with black index children showing the lowest home exposures to these microbes. The race/ethnicity variable remained a significant predictor of reduced microbial levels even after adjustment for living in an apartment and income level. These associations are most likely driven by differences in housing and living conditions by race/ethnicity that are not captured in the home characteristics questionnaire. It is likely that the relationship of ethnicity to levels of home microbial levels can be attributed to residual confounding by unmeasured housing characteristics (e.g., control over home heating) that differ by race/ethnicity. An inverse association between black race/ethnicity and endotoxin level was also observed in an earlier analysis of this birth cohort, relating endotoxin exposure in the first year of life with home/demographic characteristics (Park et al 2000, 2001a). The relationship between microbial exposure levels and SES may, at least in part, account for the higher rates of respiratory disease observed in minority populations. Because endotoxin protects against allergic disease in our cohort, the lower GNB levels in African-American households may represent unmeasured home characteristics that represent a risk factor for allergy and asthma.

Our epidemiologic approach could also have translational implications. Our main focus was not on pathogenic microorganisms that may cause clusters of acute disease. In our research we use microbial markers for epidemiologic reasons, with more of an interest in exposure to elevated levels of commensal organisms. Many commensal microbial organisms have irritant properties. However, exposure to microbial components at critical stages of life is also hypothesized to protect against allergic disease. The more specifically we identify organisms adversely or positively associated with health effects, the more we can do translational research, with the potential to identify components that might ultimately have therapeutic purposes. This is an additional motivating factor for measuring specific biomarkers in the home, rather than relying on questionnaire responses.

### Predictive value of home characteristics

We focused on the predictive power of home characteristics for levels of biomarkers above the median because we have identified health effects of exposures categorized using this cut-point (Park et al. 2001a; Litonjua et al. 2002). PPVs of home characteristics were generally poor, although dampness, infrequent cleaning, and pets predicted elevated (> median) levels of GNB in bed dust samples 71–75% of the time. However, we saw a drop in these PPVs and an increase in the NPVs when we set the threshold for elevated microbial levels at the 75th percentile (as opposed to the median). Thus, the absence of dampness, high cat allergen, or infrequent cleaning predicts the absence of higher mold or GPB at this cut-point.

To use home characteristics as exposure surrogates with minimal misclassification, both PPV and NPV values would have to be sufficiently high (≥ 90%). Although shifting the microbial exposure threshold produced increases in either PPV or NPV, these values never simultaneously reached the predictive probabilities necessary to justify the use of home characteristics as substitutes for microbial biomarker quantification.

### Limitations

Although we were able to study predictors of the complex microbial milieu at a single time point, measurement of the entire biomarker panel at additional time points was cost prohibitive, thereby limiting our ability to analyze longitudinal changes in home microbial levels. We assessed cleaning frequency in the bedroom by questionnaire, but we did not have data on specific cleaning practices (e.g., dry vs. wet mopping/dusting) that might be associated with decreasing microbial biomarker levels. Another limitation of this study is the sensitivity of predictive value calculations to changes in cut-points for “high” microbial exposure levels. For this reason, our main statistical analyses focused on microbial biomarkers as continuous outcome measures, which yielded the most stable estimate.

## Conclusions

Significant predictors of home microbial exposure encompass categories of dampness, pets, cleaning, and demographics (SES, race/ethnicity, and sex). Home characteristics partially explain variation in microbial exposure levels, but they cannot serve as surrogates or as markers of differentiation between microbes. In this U.S. urban environment, if one is seeking to evaluate health effects of specific groups of organisms that (like mold vs. GNB) may have differing effects on children (Zeldin et al. 2006), then it becomes necessary to measure those biomarkers rather than relying solely on home characteristics to profile microbial exposures in the home.

## Figures and Tables

**Table 1:** Characteristics of homes with bed and family room dust samples [*n* (%)].

Category	Characteristic	Homes with family room sample (*n* = 354)	Homes with bed dust sample (*n* = 294)
Dampness	Report of water damage in home or visible mold/mildew in room sampled (water/mold index)	112 (31.7)	88 (29.9)
	Humidifier use in room sampled	16 (4.5)	89 (30.4)
	Dehumidifier use in room sampled	13 (3.7)	1 (0.3)
	Central air	103 (29.2)	78 (26.5)
	Living in an apartment	16 (4.5)	14 (4.8)
Pets	Currently owns a dog	76 (21.5)	70 (23.8)
	Currently owns a cat	76 (21.5)	67 (22.8)
Pests	Any mouse sightings in past year	111 (31.3)	103 (35.0)
	Any cockroach sightings in the past year	10 (2.8)	3 (1.0)
Cleaning	Cleaning frequency ≥ once/week	NA	160 (54.4)
Reservoir for microbes	> Four stuffed animals on bed	NA	50 (17.0)
	Wall-to-wall carpet	113 (31.9)	108 (36.7)
SES	Income < $35,000/year	12 (3.4)	11 (3.7)
	Black race/ethnicity	26 (7.4)	26 (8.9)
Other	Continuously burning pilot light	48 (13.6)	NA

NA, not analyzed.

**Table 2:** Biomarker levels in dust.

Category	Characteristic	*n*	Mean	Median	Interquartile range
GNB	Endotoxin (LAL) (EU/mg)				
	Bed dust	294	23.7	18.9	12.3–31.3
	Family room dust	354	64.3	38.6	25.6–58.9
	Endotoxin (rFC) (EU/mg)				
	Bed dust	294	8.8	5.9	3.2–10.6
	Family room dust	354	28.7	10.3	5.3–23.9
	Midchain 3-OHFAs (pmol/mg)				
	Bed dust	294	39.6	35.8	28.0–47.2
	Family room dust	354	64.6	60.3	48.9–75.6
GPB	Muramic acid (ng/mg)				
	Bed dust	293	69.4	62.6	45.0–79.4
	Family room dust	350	77.4	72.2	54.6–95.3
Fungi	β-d-Glucan (ng/mg)				
	Bed dust	294	19.4	16.8	11.6–24.6
	Family room dust	353	29.9	25.0	17.0–36.3
	Ergosterol (ng/mg)				
	Bed dust	294	1.3	1.0	0.6–1.7
	Family room dust	344	4.1	2.5	1.7–4.1

**Table 3:** Biomarker correlations: Pearson correlation (log_10_ biomarker level).^a^

Category	Characteristic	β-d-Glucan	Ergosterol	Muramic acid	Endotoxin (rFC)	Endotoxin (LAL)	Midchain 3-OHFAs
Bed dust							
Fungi	β-d-Glucan	1.0					
	Ergosterol	0.32	1.0				
GPB	Muramic acid	0.18	0.24	1.0			
GNB	Endotoxin (rFC)	0.23	0.35	0.20	1.0		
	Endotoxin (LAL)	0.25	0.41	0.32	0.67	1.0	
	Midchain 3-OHFAs	0.42	0.43	0.26	0.39	0.45	1.0
Family room dust							
Fungi	β-d-Glucan	1.0					
	Ergosterol	0.22	1.0				
GPB	Muramic acid	0.15	0.13	1.0			
GNB	Endotoxin (rFC)	0.22	0.20	0.25	1.0		
	Endotoxin (LAL)	0.22	0.24	0.29	0.82	1.0	
	Midchain 3-OHFAs	0.23	0.28	0.35	0.42	0.39	1.0

a *p* < 0.0001 for all *r* ≥ 0.23; *p* < 0.05 for all *r* ≥ 0.10 and ≤ 0.22.

**Table 4:** Bed dust: percent difference in microbial biomarkers associated with predictors (home characteristics, demographics, and allergen levels) in multiple regression models (95% confidence interval for percent difference).^a^

Characteristic and model	GNB			GPB	Fungi	
	Recombinant factor C	LAL	Midchain 3-OHFAs	Muramic acid	Ergosterol	β-d-Glucan
Multiple regression models with questionnaire and allergen (pest/pet) predictors						

Dampness						
Water/mold	38.6 (11.1 to 72.9)*	22.9 (4.9 to 43.9)*	—	7.5 (−5.1 to 21.8)	17.7 (−1.1 to 40.1)	6.5 (−6.8 to 21.8)
Central air	—	—	−11.9 (−19.9 to −3.1)*	−11.4 (−22.0 to 0.7)	−29.3 (−41.1 to −14.7)*	−14.2 (−25.8 to −1.6)*
Apartment	—	—	—	—	—	−31.1 (−48.3 to −8.2)*
Pets						
Can f 1 ≥ 20 μg/g	16.1 (−9.4 to 48.7)	11.3 (−6.7 to 32.7)	13.7 (2.5 to 26.2)*	13.4 (−1.4 to 30.4)	—	20.6 (3.7 to 40.2)*
Fel d 1 ≥ 8 μg/g	36.9 (8.3 to 73.0)*	34.2 (13.5 to 58.5)*	—	—	—	—
Pests						
Bla g 1 detectable	—	—	—	—	−42.9 (−63.2 to −11.6)*	—
Cleaning						
≥ 1/week	−16.6 (−31.9 to 2.1)	−15.0 (−26.4 to −1.8)*	−9.3 (−17.1 to −1.8)*	−11.9 (−21.4 to −1.4)*	−15.1 (−27.6 to −0.4)*	—
Reservoir for microbes						
Wall-to-wall carpet	—	—	—	—	23.7 (4.0 to 47.1)*	—
> Four stuffed animals	—	—	13.2 (1.1 to 26.7)*	—	—	—
SES						
Black race/ethnicity^b^	−41.8 (−59.2 to −16.9)*	−18.6 (−36.8 to 5.0)	—	—	—	—
Season^c^						
Spring	20.6 (−8.5 to 32.9)	10.0 (−9.6 to 34.0)	−3.5 (−14.2 to 8.5)	21.5 (4.2 to 41.6)*	−15.1 (−31.5 to 5.2)	10.3 (−6.4 to 30.0)
Summer	3.3 (−19.7 to 32.9)	9.3 (−8.6 to 30.8)	34.5 (20.7 to 49.8)*	0.3 (−13.0 to 15.7)	8.6 (−10.9 to 32.3)	47.9 (27.1 to 72.0)*
Autumn	9.5 (−22.0 to 53.6)	3.8 (−18.4 to 32.2)	−2.2 (−15.2 to 12.9)	23.7 (2.0 to 49.9)*	−5.5 (−27.3 to 22.9)	16.6 (−4.8 to 42.7)
Model adjusted *R*^2^	7.7	7.1	19.0	5.6	9.3	12.7

Multiple regression models with questionnaire predictors						

Dampness						
Water/mold	35.1 (8.0 to 68.9)*	20.3 (2.5 to 41.3)*	—	6.4 (−5.2 to 21.9)	17.2 (−1.7 to 39.8)	6.3 (−7.2 to 21.6)
Central air	—	—	−12.2 (−20.3 to −3.4)*	−10.9 (−22.2 to 0.4)	−30.4 (−42.4 to −15.9)*	−14.5 (−25.5 to −1.9)*
Apartment	—	—	—	—	—	−29.9 (−47.4 to −6.4)*
Pets						
Dog ownership	15.6 (−9.1 to 46.9)	9.1 (−8.1 to 29.6)	8.3 (−2.0 to 19.6)	11.8 (−3.7 to 25.9)	—	15.8 (0.2 to 33.9)*
Cat ownership	18.7 (−6.7 to 51.1)	17.2 (−1.4 to 39.3)	—	—	—	—
Pests						
Cockroach sighting	—	—	—	—	1.7 (−54.0 to 125.3)	—
Cleaning						
≥ 1/week	−17.2 (−32.5 to 1.5)	−15.7 (−27.1 to −2.4)*	−9.1 (−16.6 to −1.0)*	−11.4 (−21.2 to −1.1)*	−15.3 (−27.9 to −0.5)*	—
Reservoir for microbes						
Wall-to-wall carpet	—	—	—	—	20.7 (1.4 to 43.7)*	—
> Four stuffed animals	—	—	12.7 (0.7 to 26.3)*	—	—	—
SES						
Black race/ethnicity^b^	−41.5 (−59.1 to −16.2)*	−18.3 (−36.9 to 5.7)	—	—	—	—
Season^c^						
Spring	16.1 (−11.9 to 53.1)	5.9 (−13.1 to 29.1)	−2.5 (−13.1 to 9.3)	19.3 (2.2 to 39.2)*	−16.4 (−32.7 to 3.9)	10.8 (−6.1 to 30.6)
Summer	0.7 (−21.8 to 29.6)	6.7 (−11.0 to 27.9)	34.3 (20.8 to 49.3)*	−3.8 (−16.5 to 10.8)	8.0 (−11.6 to 31.9)	48.3 (27.4 to 72.6)*
Autumn	5.1 (−25.2 to 47.6)	−0.05 (−21.7 to 27.6)	−1.8 (−14.7 to 13.1)	19.8 (−0.7 to 44.6)	−7.1 (−28.8 to 21.2)	16.0 (−5.3 to 42.1)
Model adjusted *R*^2^	6.2	4.2	18.0	7.8	9.3	12.7

a Absence of the home characteristic served as the reference category for each predictor unless otherwise noted. Dashes indicate that the characteristic was not entered into the model.

b Reference category is all other race/ethnicity classifications (white, Hispanic, Asian, and other).

c Reference category is winter.

* *p* < 0.05.

**Table 5:** Family room dust: percent difference in microbial biomarkers associated with predictors (home characteristics, demographics, and allergen levels) in multiple regression models (95% confidence interval for percent difference).^a^

Characteristic and model	GNB			GPB	Fungi	
	Recombinant factor C	LAL	Midchain 3–OHFAs	Muramic acid	Ergosterol	β-d-Glucan
Multiple regression models with questionnaire predictors						

Dampness						
Water/mold	65.6 (23.8 to 113.5)*	43.8 (20.5 to 71.5)*	—	5.6 (−4.4 to 16.7)	18.6 (−0.1 to 40.8)	9.4 (−5.2 to 26.2)
Central air	−28.2 (−44.7 to −3.4)*	−16.2 (−30.1 to 0.4)	—	−14.2 (−22.5 to −4.8)*	—	—
Apartment	10.8 (−41.2 to 107.9)	−15.0 (−43.5 to 27.9)	−15.1 (−30.5 to 3.7)	—	−39.2 (−58.5 to −11.0)*	−31.1 (−50.6 to −5.7)*
Pets						
Can f 1 ≥ 20 μg/g	21.7 (−15.5 to 56.1)	−0.9 (−18.7 to 21.0)	−2.4 (−5.2 to 18.4)	5.9 (−5.2 to 18.4)	−22.1 (−35.7 to −5.7)*	−12.1 (−25.1 to 3.1)
Fel d 1 ≥ 8 μg/g	—	—	−8.5 (−16.7 to 0.5)	—	—	—
Pests						
Bla g 1 detectable	—	—	—	—	—	−30.5 (−52.6 to 1.6)
Mouse sighting	—	—	—	−10.8 (−19.3 to −1.4)*	—	—
SES						
Black race/ethnicity^b^	−43.5 (−66.7 to −8.5)*	−6.4 (−32.5 to 29.8)	−12.1 (−25.1 to 3.2)	—	—	−22.9 (−40.5 to −0.2)*
Income < $35, 000/year	83.5 (−11.2 to 287.8)	27.0 (−21.2 to 104.7)	3.1 (−11.6 to 20.2)	—	−27.4 (−45.9 to −2.5)*	—
Number/sex of children						
Per boy	15.9 (0.5 to 33.7)*	12.5 (2.5 to 23.4)*	—	—	—	—
Per girl	—	—	—	−5.9 (−10.7 to −0.8)*	—	−8.2 (−14.8 to −0.9)*
Wall-to-wall carpet	—	−8.1 (−15.8 to 0.1)	—	—	—	—
Season^c^						
Spring	−8.3 (−34.9 to 29.0)	−6.7 (−25.2 to 16.4)	9.3 (−2.0 to 22.0)	3.8 (−8.2 to 17.4)	0.9 (−18.5 to 24.8)	1.2 (−15.3 to 20.9)
Summer	−13.3 (−37.2 to 19.8)	−10.3 (−27.2 to 10.6)	21.8 (9.7 to 35.3)*	−10.6 (−20.5 to 0.5)	16.5 (−5.1 to 43.1)	24.1 (4.8 to 46.8)*
Autumn	1.1 (−33.0 to 52.7)	−15.2 (−35.1 to 10.7)	−1.9 (−14.0 to 12.0)	−0.6 (−14.4 to 15.4)	13.2 (−12.3 to 46.0)	16.5 (−5.9 to 44.2)
Model adjusted *R*^2^	8.3	4.9	6.9	4.9	4.6	6.0

Multiple regression models with questionnaire and allergen (pest/pet) predictors						

Dampness						
Water/mold	61.3 (22.8 to 111.7)*	42.5 (19.4 to 70.0)*	—	5.1 (−4.8 to 16.1)	18.0 (−0.7 to 40.3)	12.5 (−2.4 to 29.7)
Central air	−26.4 (−44.3 to −2.7)*	−15.7 (−29.7 to 1.0)	—	−13.8 (−22.2 to −4.4)*	—	—
Apartment	10.0 (−41.4 to 106.4)	−14.7 (−43.3 to 28.4)	−15.3 (−30.6 to 3.4)	—	−38.1 (−57.5 to −9.3)*	−29.1 (−48.6 to −2.3)*
Pets						
Dog ownership	23.5 (−9.7 to 68.7)	7.2 (−12.5 to 31.3)	−8.0 (−16.7 to 1.5)	10.9 (−1.0 to 24.2)	−19.1 (−33.5 to −1.6)*	−15.1 (−27.9 to 0.1)
Cat ownership	—	—	−8.2 (−16.9 to 1.4)	—	—	—
Pests						
Cockroach sighting	—	—	—	—	—	−38.8 (−58.9 to −8.9)*
Mouse sighting	—	—	—	−10.8 (−19.3 to −1.4)*	—	—
SES						
Black race/ethnicity^b^	−44.8 (−66.7 to −8.7)*	−5.9 (−32.1 to 30.4)	−11.6 (−24.7 to 3.7)	—	—	−24.0 (−41.2 to −1.9)*
Income < $35, 000/year	85.4 (−11.2 to 287.0)	26.3 (−21.5 to 103.8)	2.6 (−12.0 to 19.6)	—	−27.4 (−46.0 to −2.4)*	—
Number/sex of children						
Per boy	16.3 (0.8 to 34.1)*	13.0 (3.1 to 24.0)*	—	—	—	—
Per girl	—	—	—	−6.0 (−10.8 to −0.9)*	—	−8.7 (−15.3 to −1.5)*
Wall-to-wall carpet	—	−7.9 (−15.6 to 0.5)	—	—	—	—
Season^c^						
Spring	1.5 (−32.7 to 53.1)	−6.4 (−25.0 to 16.9)	9.0 (−2.3 to 21.6)	4.5 (−7.6 to 18.1)	−0.3 (−19.5 to 23.5)	1.2 (−17.2 to 18.1)
Summer	−13.8 (−37.6 to 19.0)	−10.3 (−27.6 to 10.1)	22.6 (10.4 to 36.2)*	−10.9 (−20.7 to 0.2)	16.6 (−5.2 to 43.3)	22.5 (3.5 to 44.9)*
Autumn	−7.3 (−34.0 to 30.5)	−15.0 (−34.9 to 11.0)	−1.5 (−13.7 to 12.3)	−0.3 (−14.1 to 15.7)	13.4 (−12.2 to 46.4)	13.8 (−8.0 to 40.8)
Model adjusted *R*^2^	5.8	7.8	7.5	5.5	6.5	6.9

a Absence of the home characteristic served as the reference category for each predictor unless otherwise noted. Dashes indicate that the characteristic was not entered into the model.

b Reference category is all other race/ethnicity classifications (white, Hispanic, Asian, and other).

c Reference category is winter.

* *p* < 0.05.
